# Utility assessment of an Enzyme‐linked immunosorbent assay for detection of subclinical cases of caseous lymphadenitis in small ruminant flocks

**DOI:** 10.1002/vms3.297

**Published:** 2020-06-22

**Authors:** Lina Costa, Belén Huerta, Ángela Galán‐Relaño, Lídia Gómez‐Gascón, Anabela Almeida, Inês Viegas, Alfonso Maldonado

**Affiliations:** ^1^ Department of Agrarian and Veterinary Sciences Agrarian School of Elvas Polytechnic Institute of Portalegre Portalegre Portugal; ^2^ Department of Animal Health Faculty of Veterinary Medicine University of Córdoba Cordoba Spain; ^3^ Vetdiagnos Veterinary Diagnostic Laboratory Cantanhede Portugal; ^4^ ICAAM ‐ Institute of Mediterranean Agricultural and Environmental Sciences Institute of Advanced Research and Training University of Évora Évora Portugal

**Keywords:** caseous lymphadenitis, ELISA, Portugal, seroprevalence, small ruminants

## Abstract

The actual prevalence of CLA (caseous lymphadenitis) in small ruminant flocks is underestimated in many countries, and because it is not a notifiable disease, it will continue to spread without data and information about its real economic impact. The difficulty in the accurate identification of the causative agent in internal subclinical cases allows the disease to spread within and between flocks. This research intends to assess the utility of an ELISA (enzyme‐linked immunosorbent assay) test in the detection of internal subclinical cases of CLA in farms and to simultaneously add data on the seroprevalence of the disease in Portugal. Sera from 756 small ruminants, 70% sheep (528/756) and 30% goats (228/756) were screened for antibodies against *Corynebacterium pseudotuberculosis* using the ELISA technique based on a recombinant phospholipase D (ELITEST CLA # CK105A^®^). The animals showing internal lesions (*n* ꞊ 58) were sampled for the identification of the aetiological agent. In this investigation, the prevalence of CLA was 34% (258/756), with the ELISA test showing a low specificity (78%) and high sensitivity (100%). The proof was able to detect 57% (13/23) of subclinical cases of CLA confirmed by *postmortem* examination and conventional PCR (polymerase chain reaction). The results also reveal that goats have a higher propensity for the disease, and dairy farms and non‐extensive production units appear to be more susceptible to CLA. This research clarifies an actual problem and pointed out the importance of CLA in small ruminant herds in Portugal. Finally seems to demonstrate that the ELISA test is a good diagnostic tool for use in CLA eradication programmes.

## Graphical abstract

The actual prevalence for CLA (caseous lymphadenitis) in small ruminants herds is underestimated in many countries, and not being a notifiable disease, it will continues to spread without data and information about its real economic impact. This fact takes major relevance in countries like Portugal, where small ruminants enter as an important part of livestock production. The difficulty in the accurate identification of the causative agent in subclinical and visceral cases allows the disease to spread in a flock and between them. This research work pretends to assess the utility of an ELISA (enzyme‐linked immunosorbent assay) test to detect visceral and subclinical cases of CLA in the farm. And simultaneous add data on seroprevalence of the disease in Portugal.

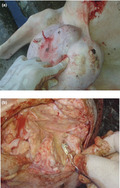

## 1 INTRODUCTION

*Corynebacterium pseudotuberculosis* is the aetiological agent of CLA (caseous lymphadenitis), a chronic bacterial disease that characteristically affects sheep and goats (Baird & Fontaine, 2007; Quinn, Markey, Leonard, Hartigan, & Fanning, 2011). This microrganism is a Gram‐positive, facultative anaerobic, intracellular rod, catalase‐positive, pleomorphic, non‐mobile and non‐sporulated classified as an actinomycete (*Actinomycetales: Corinebacteriaceae*) (Brown, Olander, & Alves,^1987^; Baird & Fontaine, 2007; Quinn et al., 2011). Considering the biotypes of *C. pseudotuberculosis*, based on the nitrate reduction capacity, nitrate‐negative strains are referred as serotype I (biotype *ovis*) and nitrate‐positive strains are classified as serotype II (biotype *equi*). Isolates from sheep and goats are usually nitrate‐negative (serotype I‐biotype *ovis*), whereas isolated strains from horses are tipically nitrate‐positive; the isolates of bovine origin are variable (Soares et al., 2013; Torres et al., 2013). The pathogenesis of *C. pseudotuberculosis* strains is related to the secrection of toxin factors such as Phospholipase D (PLD) and the lipid content of its cell walls (Torres et al., 2013). Phospholipase D it's a sphyngomyelin‐specific phospholipase that is able to dissociate sphygomyelin into phosphate and choline, and a specific exotoxin from C. pseudotuberculosis; wich is responsible for the lysis of sphyngomyelin and agent's passing. This mechanism cause plasma flow from inside the vessels to lymph nodes and for increased vascular permeability. PLD is resposible for many biologic actions such as dermo necrosis, destruction of macrophages in goat's infections, interferes with the process of chemotaxis of neutrophils in sheep and is lethal to phagocytic cells, actions that contributes to the dissemination of the agent from the point of infection to other parts of the organism (Aquino de Sá et al., 2013; Smith Bradford, 2015; Mahmood et al., 2016

The disease occurs worldwide with high prevalence values and causes serious economic losses, particularly in sheep farms and it is more evident in small producers (Al‐Gaabary, Osman, Ahmed, & Oreiby, 2010; Aquino de Sá et al., 2013; Arsenault et al., 2003; Jung et al., 2015; Malone et al., 2006; Zavoshti, Khoojine, Helan, Hassanzadeh, & Heydari, 2012). CLA is considered one of the most important diseases, in economic terms, of sheep and goats in the USA, Canada and Brazil causing significant losses in countries where the disease is considered endemic (Çetinkaya et al., 2002; Farias et al., 2019; Guimarães, 2011; Latif et al., 2016; Paton, 2003; Pavan, Robles, Cairó, Marcelino, & Pettinari, 2011; Windsor, 2014). In Australia, CLA and Johne's disease (paratuberculosis) are currently considered the two most important chronic diseases in sheep (Paton, 2010; Windsor, 2014).

This disease continues to spread worldwide, not only in small ruminants but also with pathogenic relevance in horses (Barba et al., 2015; Boysen, Davis, Beard, Lubbers, & Raghavan, 2015; Guedes et al., 2015; Spier & Azevedo, 2017), cattle (Rocha, 2011; Shpigel, Elad, Yeruham, Winkler, & Saran, 1993; Smith Bradford, 2015), wild ungulates such as the Iberian ibex (*Capra pyrenaica hispanica*) (Colom‐Cadena et al., 2014) and swine (Oliveira et al., 2014). In sheep and goats, the disease appears in two major forms, namely, external form, with the development of subcutaneous and lymph nodes abscesses, and the internal form, without any obvious clinical symptoms. The number of infected animals in a herd presenting visceral lesions can be significantly higher than those showing superficial signs of the disease (Costa, Maldonado, Huerta, & Almeida, 2019; Smith Bradford, 2015). *C. pseudotuberculosis* is a very infectious organism with the capacity to persist in the environment for several months (Almeida et al., 2016; Brown et al., 1987; Quinn et al., 2011). These facts support the difficulty in monitoring the status of disease as well as its ease in spreading.

As a non‐notifiable disease, CLA remains, in some countries, of unknown real prevalence and economic relevance. This situation is significant in countries such as Portugal, where small ruminants represent an important part of the national livestock production. Continental Portugal has a total estimate of 2,218,000 sheep and 326,000 goats, and almost half (46%) of this herd is in the study region, the province of Alentejo (1,324,000 sheep and 108,000 goats) (Statistics PORTUGAL, 2009; ). The annual production of lamb meat and certified sheep cheese has its largest representation in Alto Alentejo (Statistics PORTUGAL, 2000, 2014).

If the disease has been established in a herd, eradication of infected animals is difficult due to poor response to antibiotic therapy (Gómez‐Gascón et al., 2013; Velasco & Fernández, 2002), and difficulties in detection of infected animals (Aquino de Sá et al., 2013). The difficulty in accurately identifying subclinical cases of CLA favours the spread of the disease within and between herds. The screening and elimination of subclinical animals is an important measure in disease control programmes (Farias et al., 2019; Nassar et al., 2014). ELISA (enzyme‐linked immunosorbent assay) is an economical and simple method to perform as a routine technique in CLA diagnosis; however, ELISA tests are not being used in the control and eradication programmes of CLA, either because they present low sensitivity and specificity (Menzies, Hwang, & Prescott, 2004), have not been fully tested or have not been prepared for both species, sheep and goat (Hoelzle et al., 2013; Oreiby Atef, 2014). In large‐scale control and eradication programmes, or for the detection of animals with unapparent infection and, consequently, contributing to preventing the dispersion of the disease, ELISA would be the most suitable technique (Hoelzle et al., 2013; Menzies et al., 2004; Nassar et al., 2014; Oreiby et al., 2015; Oreiby Atef, 2014). Very few studies have evaluated the best diagnostic technique to detect subclinical cases of CLA (Barral et al., 2019), but the use of serodiagnosis as a tool for the control and eradication of CLA in goat farms has been successfully implemented in some cases (Derckeson et al., 2000; Kaba, Kutschke, & Gerlach, 2001). However, in sheep, especially in individuals with unapparent infection and internal abscesses, serological tests show low reliability due to reduced sensitivity and differences in specificity when compared to those from tests using the culture of *C. pseudotuberculosis* (Binns, Green, & Bailey, 2007).

This study aims to assess the utility of the ELISA technique using the commercial ELISA ELITEST CLA # CK105A^®^ for detection of internal subclinical cases of CLA in small ruminants. Furthermore, this field study provides data on the seroprevalence of CLA in sheep and goats living in the province of Alentejo in Southern Portugal.

## 2 MATERIALS AND METHODS

### 2.1 Animals and samples

The determination of the sample was based on the census generously provided by the regional veterinary authorities (Division of Veterinary Intervention of Portalegre—Ministry of Agriculture and Food—Portuguese Government). The objective population is made up of a total of 1,208 sheep farms (with an average of 134.8 animals per farm) and 107 goat farms (with an average of 41.8 animals per farm) (Statistics PORTUGAL, 2017a, 2017b, 2017c). In this research, we selected a field population of 82 (6%) flocks of small ruminants (55 sheep and 27 goat flocks), from which 756 blood samples (*n* ꞊ 756) of sheep and goat were randomly collected (approximately 10 samples per holding). The procedure was carried out on the farm and in a regional slaughterhouse in the province of Alentejo in Southern Portugal.

Blood samples were collected by jugular venipuncture in vacutainer tubes without anticoagulant (Becton ‐ Dickinson). These samples were centrifuged at 5,000 rpm for 10 min to obtain blood serum, which was frozen (−20°C) in tubes (Eppendorf) until testing.

In the group of animals sampled, those showing macroscopic lesion characteristics of CLA were labelled, matching the blood sample to the collected lesion sample (*n* ꞊ 58), and subsequently preserved by freezing (−20°C) (Table 1). The farms under study are of different types of production, intensive, semi‐extensive and most extensive, characteristic of the south of the Iberian Peninsula (Table 2).

**TABLE 1:** Samples description

	Sheep number (%)	Goats number (%)	Total
Farms	55 (67.07)	27 (32.92)	82
Blood samples	528 (69.8)	228 (30.8)	756
Purulent/pyogranulomatous lesions samples	54 (93.1)	4 (6.9)	58
Number of animals/farms		Between 10 and 1,200 animals	
Average of animals per holding		114	

**TABLE 2:** Farms description

	Sheep farms number (%)	Goat farms number (%)	Total number (%)
Meat farms	50 (69.4)	22 (30.5)	72 (87.8)
Dairy farms	7 (70)	3 (30)	10 (12.2)
Total			82 (100)
Extensive farms	55 (70.5)	23 (29.48)	78 (95.1)
Non‐extensive farms (semi‐extensive and intensive)	2 (50)	2 (50)	4 (4.87)
Total			82 (100)
Small‐scaled farms (<50 animals)			51 (62.2)
Medium‐sized farms (>50 and <200 animals)			17 (20.7)
Large‐scaled farms (>200 animals)			14 (17.1)
Total			82 (100)

The farms were classified according to size in the categories small (<50 animals), medium (>50 and <200 animals) and large (>200 animals). In this study, approximately 60% of the farms are small‐scaled, 20% are medium‐sized and 17% are large‐scaled (Table 2). The distribution by species among the sampled farms is like the total of animals: 70% are sheep farms and 30% are goat herds (Table 3).

**TABLE 3:** Animals description

	Sheep number (%)	Goat number (%)	Total number (%)
Meat animals	459 (70)	196 (29.9)	655 (86.6)
Dairy animals	71 (70)	30 (29.7)	101 (13.4)
Total			756 (100)
Extensive regime	500 (69.9)	215 (30)	715 (94.6)
Non‐extensive regime (semi‐extensive and intensive)	29 (70.7)	12 (29.2)	41 (5.4)
Total			756 (100)

Of the total sampled animals, 87% (655/756) are from meat production units and 13% (101/756) are from milk farms; 94% (715/756) of the animals were raised under extensive production, 3% (21/756) were raised under the intensive regime and 3% (20/756) were raised under the semi‐extensive regime, which means that 6% (41/756) were raised under the non‐extensive regime (Table 2).

### 2.2 ELISA technique

In this work, we use ELITEST CLA # CK105A^®^, a commercial enzyme immuno‐assay (EIA), for the detection of IgG antibodies specific for the causative agent of caseous lymphadenitis in sheep or goat sera. This test is a direct ELISA that uses a recombinant phospholipase D (PLD), an important virulence factor of *Corynebacterium pseudotuberculosis*, to detect anti‐PLD IgG antibodies in sera from sheep and goats with CLA (HYPHEN BioMED, 2007) PLD is not known to be produced by any other sheep pathogenic bacteria, rendering it a very specific test (HYPHEN BioMed, 2007).

The results were evaluated using an ELISA microtiter reader (BioTek EL × 808, United States) according to the manufacturer's instructions.

According to the manufacturer's information, the ELITEST CLA # CK105A^®^ presents a specificity of 98% for sheep and goats and a sensitivity of 94% and 79% for goats and sheep, respectively.

### 2.3 Conventional PCR assay

Methodology and results of the conventional PCR assay made for the identification of *C. pseudotuberculosis* in pyogranulomatous lesions and for the cross‐sectional study to assess the performance of the ELISA test was developed as described by Pacheco et al. (2007), Costa et al. (2019) and Pacheco et al. (2007).

### 2.4 Statistical analysis

The ELISA assay results were subjected to frequency analysis (prevalence of the disease). The same method was applied to the data regarding the species, productive aptitude, production regime and dimension (size) of the herd. The performance of the ELISA test was evaluated by association tests, namely, *χ* ^2^ (Chi‐square—Fischer's Exact Test) with a 95% confidence interval. The statistical analysis was performed with SPSS (Statistical Package for Social Sciences) 22.

## 3 RESULTS

In this investigation, we found a prevalence of CLA of 34% (258/756) (Table 4), with the ELISA test showing a low specificity, 78% (35/45) and high sensitivity, 100% (13/13) (Table 5).

**TABLE 4:** Prevalence values

Prevalence	
(within ELISA who also did PCR)	22.4%
(all ELISA)	34.1%

**TABLE 5:** ELISA and PCR results

		PCR		Total
		Positive	Negative	
ELISA	Positive	13	10	23
	Negative	0	35	35
Total		13	45	58
False negatives number (%)		0 (0%)		
False positives number (%)			10 (43%)	

The descriptive analysis of the results suggests that the proportion of disease higher in goats, and such increased propensity is confirmed by the statistical test (*χ* ^2^ (1, *N* ꞊ 756) ꞊ 30.773, *p* < .01) (Table 6).

**TABLE 6:** ELISA results

	ELISA negative number (%)	ELISA positive number (%)	Total	Fischer's exact test
Sheep	381 (72.15)	147 (27.8)	528	*χ* ^2^ (1, *N* ꞊ 756) ꞊ 30.773, *p* < .01
Goat	117 (51.3)	111 (48.68)	228	

	ELISA negative number (%)	ELISA positive number (%)	Total	Fischer's exact test
Meat	450 (68.7)	205 (31.29)	655	*χ* ^2^ (1, *N* ꞊ 756) ꞊ 17.458, *p* < .01
Dairy	48 (47.52)	53 (52.47)	101	

	ELISA negative number (%)	ELISA positive number (%)	Total	Fischer's exact test
Extensive regime	485 (67.83)	230 (32.16)	715	*χ* ^2^ (2, *N* ꞊ 756) ꞊ 22.561, *p* < .01
Non‐extensive regime (semi‐extensive and intensive)	13 (31.7)	28 (68.29)	41	

	ELISA negative number (%)	ELISA positive number (%)	Total	
Farm level analysis	16 (19.51)	66 (80.48)	82	80% positive holdings
Small farms (<50 animals)	No reported disease			
Medium farms (>50 and <200 animals)	Farms without the reported disease have an average of 85 animals			
Large farms (> 200 animals)	Farms with the confirmed disease have an average of 171 animals (*p* < .001)			
Seroprevalence	258/756 (34%)			

Regarding productive aptitude, dairy farms appear to be more susceptible to CLA (χ2 (1, *N* ꞊ 756) ꞊ 17.458, *p* < .01); the same propensity appears to exist regarding the production regime, with the farms in the non‐extensive regime (intensive and semi‐extensive, approximately 6% of the total sample) showing an increased propensity for the disease (*χ* ^2^ (2, *N* ꞊ 756) ꞊ 22.561, *p* < .01) (Table 6).

**FIGURE 1:**
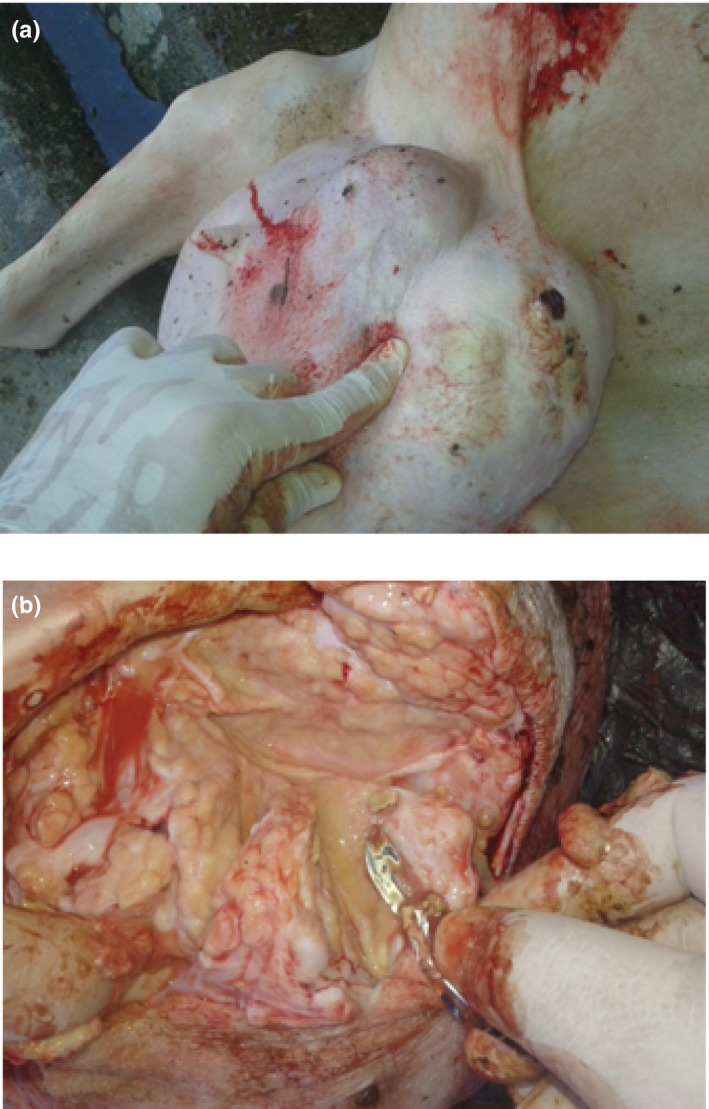
CLA in the mammary gland in dairy sheep. (a) Presence of mammary abscess. (b) Purulent material when cutting the udder

The presence of the disease in milk farms (especially sheep farms) was evident in the visceral form, namely, in the mammary glands, with identification of *C. pseudotuberculosis* performed by conventional PCR assay in this investigation (Figure 1).

When the results of the ELISA are analysed regarding the size of the farm, the data are also statistically significant. This data suggests that farms without the reported disease have an average of 85 animals, whereas farms with confirmed disease have an average of 171 animals (*p* < .001). This observation appears to mean that CLA is more prevalent in medium to large farms (50–200 animals and >200 animals) (Table 6). Although the sample is not proportionate regarding larger farms, these results seem to be relevant.

By performing a herd‐level analysis (considering an infected farm with at least one animal positive for the ELISA), of the 82 farms in the study, 66 had at least one positive animal, leading to a value of 80% (66/82) of affected holdings (Table 6).

The performance of the ELISA test technique (ELITEST CLA # CK105A^®^) was evaluated by determining the positive predictive value (PPV) and negative predictive value (NPV). In this analysis, the ELISA test allowed the detection of 57% of subclinical animals, which was confirmed by the *postmortem* examination and conventional PCR assay performed in pyogranulomatous lesions. In the group of animals in which both tests were performed (PCR and ELISA) (*n* ꞊ 58), 57% of the animals positive for the ELISA test were positive for the PCR assay (13/23), and all the negatives, 100%, in the ELISA test were negative for the PCR assay (35/35) (Table 7).

**TABLE 7:** ELISA performance

Sensitivity	100%
Specificity	78%
Positive predictive value (PPV)	57%
Negative predictive value (NPV).	100%

## 4 DISCUSSION

The present study shows that CLA is a prevalent disease in small ruminants in Portugal, with 34% seroprevalence. This prevalence is very close to values found in other studies, such as in Spain (26%–73% in different provinces of Andalusia, 38% in Andalusia and 80%–90% in Aragon and Navarre) (Cubero, Gonzalez, Martín, & León, 2002; Gómez‐Gascón et al., 2013), and Canada (from 152 goats subjected to necropsy, 54 (35.5%) had at least 1 abscess,*Corynebacterium pseudotuberculosis* was isolated in 37 of the 54 (68.5%) goats with abscesses, confirming that CLA was the most frequently diagnosed disease in this study, affecting 24.3% of the animals sacrificed) (Debien et al., 2013).

In recent research (Costa et al., 2019), the authors found an overall prevalence of CLA of 17% in affected small ruminant herds (with macroscopic observation of characteristic lesions in 58/335, and conventional PCR assay in 10/58, made in pyogranulomatous lesions). Smith estimated the prevalence in large breeding operations in endemic areas to be between 5% and 10% (Smith Bradford, 2015). With these data, we consider CLA an endemic disease in the Portuguese region of Alentejo.

The results show a statistically significant difference when analysing the proportion of disease between sheep and goats, and generally, goats appear to have a higher propensity for the disease. It would be important to validate these data internally and include a large number of samples of goats to properly evaluate these results.

The greater propensity for the disease in both dairy farms and farms in non‐extensive (intensive and semi‐extensive) production regimes, when compared to meat production units and extensive farms, is statistically significant (*p* < .01). This fact is due to the easier dispersion of the disease when animals are more confined, as described in the literature (Smith Bradford, 2015).

The internal form of the disease is a recent concern in milk goat farms, and *C. pseudotuberculosis* must be considered for routine bacteriological examination of milk from dairy goats (Nabih, Hussein, El‐Wakeel, El‐Razik, & Gomaa, 2018) as the presence of CLA in dairy herds can have serious consequences on the milk yield of the herd by spreading to a large number of animals in production.

The data suggest that the larger the farm is, the more predisposed it is for the disease; CLA is more prevalent in medium to large farms (50–200 animals and >200 animals, respectively), again confirming that the dissemination of the agent increases as the possibility of contact between infected animals increases.

These proportions must be carefully interpreted since they originate from a very unbalanced sample (low number of goats, low number of dairy farms and low number of farms in a non‐extensive production regime).

At the farm level, 80% (60/82) of the farms had at least one seropositive animal; these values are similar to those found in the Canadian study (Debien et al., 2013) in which eleven of the 13 farms (84.6%) analysed had at least one affected animal with CLA. A recent study revealed a very equal prevalence of CLA at the farm level (Farias et al., 2018), where 88.5% (193/218) of the investigated Brazilian herds had at least one seropositive goat infected with *C. pseudotuberculosis*.

Assessment of the utility of the ELISA test was made by comparing the results obtained with those of a conventional PCR assay. The choice of PCR instead of the gold‐standard identification technique, bacterial isolation of *C. pseudotuberculosis*, is influenced by financial (economic constraints) and practical reasons (mainly conservation and sample processing) but is mostly based on several studies that found that bacterial culture resulted in a low isolation rate and that conventional PCR was a more sensitive and specific test for CLA, with more accurate detection of the aetiologic agent (Baird & Fontaine, 2007; Costa et al., 2019; Debien et al., 2013; Kumar, Tripathi, Kumar, Sonawane, & Dixit, 2013; Oreiby et al., 2015).

The ELISA test performed in this investigation used the *Corynebacterium pseudotuberculosis* virulence factor phospholipase D (PLD), a recombinant protein that proved to be very accurate for the serodiagnosis of caseous lymphadenitis in goats and sheep (Barral et al., 2019). The ELISA test performed in this research was able to detect 57% of infected animals. These results suggest that the ELISA test is very accurate in its ability to detect subclinically infected animals. With the ELISA test detecting approximately a proportion of 0% of false‐negative animals and 43% of false‐positive animals, the use of this diagnostic technique to detect infected animals and the elimination of the herd could be a good measure to implement in small ruminant farms. Real negative animals can stay in the herd with no risk of agent persistence and dispersion, and the majority (57%) of positive infected animals will certainly be eliminated or separated from the herd core until subsequent screening or culling.

## 5 CONCLUSION

The facts found in this research lead us to the conclusion that the ELISA test may be detecting a valid proportion of subclinically infected animals, and based on these data, the authors consider the ELISA test to be a good tool for application in CLA eradication programmes in Portugal.

The eradication of a disease with the characteristics of CLA is a challenge that involves decision‐making at various levels. Factors that make eradication a challenge include the weak response of the agent to antibiotic therapy, its great ability to survive in the environment and the difficulty of detection of infected animals, the main factor responsible for the maintenance of the disease in a herd, as a source of infection.

New studies on CL prevalence in animals and herds should be conducted in the region and in the country, using serological diagnostic techniques, for the detection of asymptomatic animals. Assessment of the real damage caused by the presence of infection in small ruminant's farms and the economic impact of the disease in Portugal is needed. These data will allow to assess the need to implement control and eradication measures of the disease, as has already been done in other parts of the world.

## CONFLICT OF INTEREST

The authors declare that there is no conflict of interest regarding the publication of this paper.

## AUTHOR CONTRIBUTION

**Lina Costa:** Data curation; Funding acquisition; Investigation; Methodology; Project administration; Resources; Writing‐original draft; Writing‐review & editing. **Belén Huerta:** Formal analysis; Supervision; Validation; Visualization. **Ángela Galán‐Relaño:** Visualization. **Lídia Gómez‐Gascón:** Visualization. **Inês Viegas:** Data curation; Methodology; Software; Visualization. **Anabela Almeida:** Data curation; Investigation; Resources; Software; Visualization. **Alfonso Maldonado:** Conceptualization; Formal analysis; Investigation; Methodology; Project administration; Resources; Supervision; Validation; Visualization.
